# Amino acid–derived defense metabolites from plants: A potential source to facilitate novel antimicrobial development

**DOI:** 10.1016/j.jbc.2021.100438

**Published:** 2021-02-19

**Authors:** Anutthaman Parthasarathy, Eli J. Borrego, Michael A. Savka, Renwick C.J. Dobson, André O. Hudson

**Affiliations:** 1 Rochester Institute of Technology, Thomas H. Gosnell School of Life Sciences, Rochester, New York, USA; 2 Biomolecular Interaction Centre and School of Biological Sciences, University of Canterbury, Christchurch, New Zealand; 3 Bio21 Molecular Science and Biotechnology Institute, Department of Biochemistry and Molecular Biology, University of Melbourne, Parkville, Victoria, Australia

**Keywords:** plants, plant defense, amino acids, secondary metabolites, antibiotic resistance, AMR, antimicrobial resistance, CHIKV, chikungunya virus, CRP, cysteine-rich peptide, DENV, dengue virus, HCV, hepatitis C virus, HLP, hevein-like peptide, IAV, influenza A virus, IBV, infectious bronchitis virus, JEV, Japanese encephalitis virus, MAPK, mitogen-activated protein kinase, MRSA, methicillin-resistant *Staphylococcus aureus*, NPAA, nonproteinaceous amino acid, PA, phytoalexin, PP, phenylpropanoid, ROS, reactive oxygen species, WNV, West Nile virus, ZIKV, Zika virus

## Abstract

For millennia, humanity has relied on plants for its medicines, and modern pharmacology continues to reexamine and mine plant metabolites for novel compounds and to guide improvements in biological activity, bioavailability, and chemical stability. The critical problem of antibiotic resistance and increasing exposure to viral and parasitic diseases has spurred renewed interest into drug treatments for infectious diseases. In this context, an urgent revival of natural product discovery is globally underway with special attention directed toward the numerous and chemically diverse plant defensive compounds such as phytoalexins and phytoanticipins that combat herbivores, microbial pathogens, or competing plants. Moreover, advancements in “*omics*,” chemistry, and heterologous expression systems have facilitated the purification and characterization of plant metabolites and the identification of possible therapeutic targets. In this review, we describe several important amino acid–derived classes of plant defensive compounds, including antimicrobial peptides (*e.g.*, defensins, thionins, and knottins), alkaloids, nonproteogenic amino acids, and phenylpropanoids as potential drug leads, examining their mechanisms of action, therapeutic targets, and structure–function relationships. Given their potent antibacterial, antifungal, antiparasitic, and antiviral properties, which can be superior to existing drugs, phytoalexins and phytoanticipins are an excellent resource to facilitate the rational design and development of antimicrobial drugs.

Antimicrobial resistance (AMR) is one of the greatest global challenges of the 21st century (1). The seriousness of the AMR crisis was accentuated in 2017 by the case of a patient who succumbed to infection after the failure of all 26 United States–approved antibiotics (https://www.scientificamerican.com/article/woman-killed-by-a-superbug-resistant-to-every-available-antibiotic/). Overuse of antibiotics, their abuse in animal husbandry, and the lack of financial incentives for antimicrobial development have all contributed to the growing AMR medical threat (2, 3, 4, 5, 6). The deficiency of available antimicrobials is not limited to only those that treat bacterial infections. Fungi from the genera *Candida* and *Aspergillus* are featured in the Centers for Disease Control and Prevention list of leading threats for nonviral infections (7). In addition, an emerging fungal pathogen, *Candida auris,* was simultaneously detected on several continents and has raised concerns over the potential for climate change to spread new diseases (8). It is unfortunate that there are even fewer antifungal drugs currently available or under development than antibiotics. Furthermore, zoonotic viruses, such as SARS-CoV2, and parasitic diseases are spreading owing to disturbances from deforestation, wildlife hunting, migration, and urbanization as human populations continue to grow (9, 10, 11, 12, 13). Climate change will also facilitate infectious diseases reaching even cold, high-latitude countries (14), and current estimates projected to expose an additional 1 billion people to vector-borne diseases (15, 16, 17). As infectious disease threats accelerate globally, it is imperative to discover and develop safe and effective pharmacological treatments. The chemically diverse secondary metabolites produced by plants are potential sources to facilitate exploration, research, and development of antimicrobial compounds.

Of interest, phylogenetically related medicinal plants in geographically disconnected areas contain compounds with similar pharmacological activities and the chance of finding bioactive molecules within identified groups of medicinal plants is much greater than from randomly chosen plants (18). Approximately 70% of current drugs are derived from natural products from microbial or plant origins (19, 20). Among 1328 approved drugs between 1981 and 2016, 359 were purely synthetic, 326 were peptides and antibodies, 94 were vaccines, and 549 were nonpeptide natural products (19, 20). Screens identifying bioactive molecules have an increased likelihood of finding “hits” with natural products compared with combinatorial chemical libraries, since natural products evolved with “privileged structural motifs” promoting biological activity and useful properties, such as the tendency of natural products to enter cells *via* transmembrane transporters rather than by passive diffusion (21, 22). Although microbes undeniably possess more diverse secondary metabolism than plants, only a small fraction of the environmental microbiota are culturable (23), substantially challenging the isolation and characterization of unknown microbial compounds. Thus, more than 133,000 natural product compounds are derived from plants, compared with less than 40,000 from microbes (24). Low cost, biocompatibility, effectiveness, and well-developed analytical chemistry pipelines (25, 26) make phytochemicals an attractive source for lead development of both antimicrobial agents and for identifying compounds that aid the biological activity of existing drugs.

Phytochemicals may also be utilized to indirectly reduce AMR, for example, by supplanting antibiotics as growth promoters in animal husbandry (27) and with the incorporation of plant extracts and oils in materials designed to limit biofilm formation (28, 29, 30, 31). In addition, several studies have examined the effectiveness of plant extracts and oils that are endowed with antimicrobial properties, either alone or in combinations with existing drugs (32, 33, 34, 35, 36, 37, 38, 39, 40). Pharmacological synergy of antibiotics is a burgeoning field; for example, isoflavonoids were successfully applied against methicillin-resistant *Staphylococcus aureus* (MRSA) as antibiotic adjuvants (36). Plant-derived compounds can also be employed in novel approaches to indirectly combat bacterial infections (41, 42), by reducing their antibiotic resistance capabilities. For example, recent high-throughput natural product screens aided the identification of efflux pump inhibitors from plants (41, 43, 44). These pumps are transporter proteins used by bacteria to shuttle antibiotics out of their cells and are the major mechanisms behind multidrug-resistant strains. The antimicrobial activity of phytochemicals also extends to other kingdoms (45, 46). Natural products such as polyketides, terpenoids, alkaloids, peptides, and phenylpropanoids metabolites have provided many antifungal and antiparasitic drug leads in the last decade (47, 48). Antiviral molecules are also frequently found among purified plant compounds and herbal extracts used in traditional medicine (49) and may serve as a resource for a “Viral Toolbox,” a collection of naturally preselected scaffolds for informing antiviral development. Compared with vaccines or other biologics, which are often more expensive to manufacture, are harder to store, and require intravenous administration, relatively stable small molecules can be efficiently mass produced, stored more readily, and often administered orally. These characteristics are particularly desirable in the remote and resource-limited areas of the world. A robust repertoire of small molecule antimicrobial leads will serve an effective strategy to prepare for the inevitability of future pandemics of both humans and animals.

Lead optimization is a key step in drug development where the chemical structure of a candidate compound serves as a starting point for modifications aimed to improve the potency, selectivity, or other pharmacological parameters. Recent advances in synthetic chemistry permit the biomimetic generation of natural product structures. These advances facilitate methods to access plant polyketides and strained cyclic terpenoids that previous methods could not easily synthesize (50, 51, 52, 53).Chemoenzymatic methods have enabled the synthesis of a variety of oxygenated terpenoids (54), and breakthroughs in peptide synthesis using flow chemistry facilitate the rapid production of longer peptides than earlier methodologies (55, 56). In addition, bacterial fermentations and engineered cocultures of *Escherichia coli* with other bacteria and yeast can now produce plant-derived secondary metabolites such as alkaloids and phenylpropanoids (57, 58). These biological methods offer a great opportunity to produce rare molecules and complex structures containing stereocenters (59). Thus, the potential to produce tailored variations of major natural products families is now greater than ever before. In addition, emerging biotechnologies have been optimized for plants in recent years, including CRISPR-based gene editing (60, 61, 62), metabolomics (63), and heterologous yeast platforms (58, 64, 65, 66, 67), enabling production of diverse and complex plant compounds.

Virulence factors involved in plant and animal pathogenesis have a significant degree of overlap (68); however, a fundamental concept of plant pathology is that most plants are resistant to most pathogens. Since plants do not have circulating immune cells, a major component of their resistance is the production of potent antimicrobial secondary metabolites known as phytoanticipins and phytoalexins (PAs) (69, 70), and these small molecules are ideally suited for discovering new antimicrobial leads and antibiotic enhancers (71). PAs *sensu stricto* refer to any plant secondary metabolite that has antimicrobial properties and are produced during the defense response, whereas phytoanticipins are produced by healthy plants under natural conditions and their levels increase during stress. A clear separation between PAs and phytoanticipins is not always possible, so for the purposes of this review they are treated as the same. PAs are best characterized in economically important crop plants such as those belonging to the Brassicaceae, Fabaceae, Poaceae, Solanaceae, Triticeae, and Vitaceae families (72). Many PAs such as alkaloids, phenylpropanoids, and some sulfur-containing compounds are derived from amino acid precursors (73). In addition to these small molecule PAs, plants produce several classes of antimicrobial peptides as part of their defense response (74, 75, 76, 77). PAs and defense peptides typically target specific biological processes and are used by plants to fight microbial infection, to deter feeding by herbivores, and during competition against other plants (78, 79). These amino acid–derived defense compounds represent privileged scaffolds, which evolved to bind biological targets, and can therefore provide a rich resource for the development of antimicrobials. Here, we describe the biosynthesis of selected amino acid–derived small molecules and peptides, and their potential in the development of antimicrobials, namely, antibacterial, antifungal, anti-parasitic, and antiviral therapeutics, by focusing on structural and mechanistic aspects.

## Antimicrobial peptides

Plants produce a variety of defensive antimicrobial peptides, many of which are cysteine-rich peptides (CRPs), such as cyclotides, defensins, knottins, snakins, and thionins (74, 75, 76, 77, 80, 81). Here, we confine the discussion of the biomedical applications of compounds with a molecular mass ≤7 kDa and thereby exclude small defense proteins such as puroindolines and lipid transfer proteins. A summary of the activities of different antimicrobial peptide classes is shown in Table 1. Antimicrobial peptides are considered especially good drug leads (94) because their properties combine the advantageous chemistry of small molecules with the improved specificity of larger biologics such as antibodies (95). They are especially promising candidates for antifungal development (96).

**Table 1:** A summary of selected examples of the *in vitro* and *in vivo* antimicrobial activity of plant-derived AMPs discussed in this article

AMP family	Prominent examples (plant sources)	Activity (putative mechanism)	Type of testing, target pathogen/infection, reference	Available production methods
Cyclotides	CyO2 (*Viola odorata*)	Antibacterial (membrane binding), antifungal (membrane disruption, spore penetration), antiviral (disruption of viral integrity, pore formation in infected cells)	Animal tests, *S. aureus* wound infections (82); Mammalian cell culture, HIV-1 (83)	Chemical, chemoenzymatic, heterologous
Defensins	RsAFP2 (*Raphanus sativus*)	Antifungal (reactive oxygen species, elevated septin and ceramide, apoptosis induction; targets cell wall and membrane sphingolipids)	Animal tests (prophylactic), *Candida* spp. (84)	Chemical, heterologous
Thionins	CaThi (*Capsicum annuum*)	Antibacterial (membrane disruption), antifungal (membrane disruption, apoptosis, reactive oxygen species)	*In vitro*, bacteria and *Candida* spp. (85, 86, 87, 88)	Chemical, heterologous
Knottins	As1 (*Alstonia scholaris*)	Antiviral (inhibits viral spike protein and maturation protein)	Mammalian cell culture, influenza B virus (89)	Chemical, heterologous
Snakin-like peptides	Snakin-Z (*Zizyphus jujube*)	Antibacterial and antifungal (membrane disruption by pore formation)	Mammalian cell culture, *S. aureus* (90)	Chemical, heterologous
α-Hairpinin-like peptides	EcAMP1 (*Echinochloa crus-galli*)	Antifungal (binding cell wall carbohydrates and membrane lipids)	*In vitro*, *Fusarium* spp. (91)	Chemical, heterologous
	Luffin P1 (*Luffa cylindrica*)	Antiviral (binds the *rev* response element)	Mammalian cell culture, HIV-1 (92)	
Hevein-like peptides	(*Pereskia bleo*)	Antifungal (chitin assembly inhibition, membrane disruption)	Mammalian cell culture, *C. albicans* and *C. tropicalis* (93)	Chemical, heterologous

### Cyclotides

Plants from the seemingly unrelated Cucurbitaceae, Fabaceae, Rubiaceae, Solanaceae, and Violaceae families produce antimicrobial and insecticidal “mini-proteins,” known as cyclotides (97), which represent the best-known plant antimicrobial peptides and display activity against multiple groups of pathogens. Antimalarial activity has been reported from cyclotide-rich extracts of *Oldenlandia affinis* (Rubiaceae), a West African medicinal plant (98). Various cyclotides from the sweet violet (*Viola odorata*) demonstrated broad-spectrum antibacterial and antifungal activities, with low inhibitory concentrations against *E. coli*, *Klebsiella pneumoniae*, *Pseudomonas aeruginosa*, *Salmonella enterica*, *S. aureus,* and the fungi *Candida albicans* (99, 100), *Fusarium graminearum* (101), and *Fusarium oxysporum* (102). In Gram-negative bacteria, the interaction with phosphatidylethanolamine-lipids appears to determine species selectivity (100). In mouse models, the cyclotide cycloviolacin 2 limits subcutaneous *S. aureus* infections in surgical wounds without toxicity to monocytes while stimulating immune cell phagocytosis (82).

Several cyclotides have also been explored for their antiviral properties especially toward HIV (103). The cyclotide cycloviolacin 2 induces pore formation in HIV-infected T cells and monocytes, disrupting viral integrity and improving the efficacy of antiretroviral drugs (83, 104). It is important to note that cyclotide cycloviolacin 2 is effective at a nanomolar concentration, which is considered a safe dose for preclinical animal testing for HIV (105). Initial tests in murine models with intravenous cyclotide cycloviolacin 2 administered at < 2 mg/kg could not find any appreciable toxicity or hemolysis (106, 107).

Cyclotides usually range in size from 25 to 40 amino acids and feature a unique head-to-tail macrocyclic structure containing cystine knots that confer proteolytic stability (108, 109). They tolerate high sequence variation in the nonconserved cysteine residues and can pass through membranes, a useful quality for oral formulations. This class of antimicrobial peptides are also candidates for the modulation of protein–protein interactions, and their potential in drug development has gained attention in recent years (110). Cyclotides of the Möbius and bracelet types contain well-defined hydrophilic and hydrophobic patches, leading to an amphiphilic property similar to that of classical antimicrobial peptides (105). However, the variation of these hydrophobic patches differs among individual cyclotides, resulting in different membrane-binding properties for each (111). Owing to their short peptide lengths, cyclotides are amenable to synthesis and bioengineering efforts, which has accelerated development of synthetic analogs as antivirals (112). In addition to the chemical routes for the synthesis of cyclotides, large-scale heterologous production is reasonably achievable, since the enzymes involved in their cyclization are characterized (113).

### Defensins

Members of this group of antimicrobial peptides are usually of 45 to 54 amino acids and positively charged CRPs. They have eight Cys residues with four disulfide linkages stabilizing their triple-stranded β-sheet and α-helical regions (114, 115). Defensins are widely distributed in plant families, including many crops where they accumulate in a tissue-specific manner (74, 116, 117). Defensins bind sphingolipids (118), a promising target for a newer generation of antifungals (119). Sphingolipids are widely distributed in eukaryotes, including fungi, but are rarer and less diverse in bacteria (120). This might explain why most defensins have antifungal rather than antibacterial properties. Of importance, some plant defensins, such as DmAMP1, HsAFP1, and RsAFP2, were shown to have increased activity against clinical pathogens such as *Aspergillus flavus, C. albicans*, *Candida krusei*, and *Fusarium solani* compared with commonly used azole-derived antifungals (121).

The rice (*Oryza sativa*) defensin OsAFP1 kills *C. albicans* by inducing apoptosis and targeting cell-wall components; mutational analysis suggests that about 10 residues at the N and C termini are important for this activity (122). The defensin PsD1 from pea (*Pisum sativum*) inhibits growth of several species by interacting with sphingolipids on the fungal envelope and permeabilizing cell membranes leading to growth arrest (123). Of interest, recombinant protein analysis showed that addition of four extra amino acids at the N terminus decreased the activity of PsD1 against *Aspergillus niger* and *F. solani* by 5-fold, but not against *Neurospora crassa*. This suggests that defensins are not merely cytotoxic and instead target distinct biological functions. In fact, analysis of the mode of action of the plant defensin NaD1 showed that the presence of the fungal cell wall is essential for the antifungal effect (124). Defensins engineered to have species-specific activity would potentially be able to target pathogenic fungi while preserving beneficial fungi.

Some defensins such as HsAFP1 can also impair the fungal cell cycle independently from their antifungal activity (125) and have broad-spectrum fungicidal properties *via* distinct modes of action. The radish (*Raphanus sativus*) defensin, RsAFP2, was shown to bind fungal glucosylceramide sphingolipids but not those from plant or humans (126). Most promising, RsAFP2 was also shown to be prophylactically effective *in vivo* in mouse models against candidiasis (84). Recent work shows that it does not induce membrane permeabilization but instead triggers reactive oxygen species (ROS) production (127), increased septin and ceramide levels (128), and ultimately apoptosis without caspase activation (129). RsAFP2-mediated fungal inhibition can synergize with the antifungal drug caspofungin preventing *C. albicans* biofilm formation (130). *C. albicans* biofilms tolerate common antifungals as well as the human immune system extremely well (131), making the synergistic biofilm inhibition an important advancement.

### Thionins

This class of antimicrobial peptides contains positively charged CRPs ∼5 kDa in size. Their structure consists of antiparallel α-helices and a double-stranded β-sheet with three to four disulfide bridges. They are classified into five groups of α-/β-thionins with high homology and previously included the superficially similar γ-thionins, now known as defensins (80, 132). Most thionins have a groove between the α-helices and β-sheets with a conserved Tyr residue and may lead to cell lysis through membrane leakage (133). CaThi is a thionin isolated from the fruit of jalapeño (*Capsicum annuum,* Solanaeceae) and active against both fungi and bacteria (85). Of interest, although CaThi caused membrane disruption in six *Candida* species, nuclear localization and ROS production were observed only in *C. tropicalis* (86). It also exhibited synergistic effects with the common azole antifungal fluconazole, making *F. solani* susceptible to low concentrations of the antifungal (87) and inhibiting all six *Candida* species tested (86). It was shown to induce apoptosis in *C. tropicalis* by caspase and pH imbalance–related mechanisms (88).

### Knottins

These AMPs contain three disulfide linkages, whereby a pair of disulfides form a loop through which the third disulfide bond passes, creating a heat-stable and protease-resistant structure known as an inhibitor cystine knot (134). Knottins often possess protease inhibitory activities at nanomolar concentrations and occur in the seeds of several plants, such as MJTI I and II in the garden four o'clock (*Mirabilis jalapa*), MCoTI-III in bitter gourd (*Momordica cochinchinensis*), EETI-III in squirting cucumber (*Ecballium elaterium*), and SOTI-III in spinach (*Spinacia oleracea*) (135, 136, 137). Cystine knot α-amylase inhibitors, which are approximately 30 amino acid–long knottins produced by the amaranthaceae and apocynaceae families, contain one or more *cis*-proline bonds (138, 139, 140). Cystine knot α-amylase inhibitors–type knottins from the blackboard tree *Alstonia scholaris* called alstotides were demonstrated to be cell-permeable inhibitors of the infectious bronchitis virus (IBV) and dengue virus (89). One of the alstotides, As1, was shown to rapidly bind and block the function of the IBV spike (S) protein, which drives viral fusion with the cell membrane. This activity is reduced when the N terminus is blocked by biotinylation (89) highlighting the importance of the N terminus and its neighboring residues for the antiviral activity of alstotides. Pull-down assays show that As1 also binds to the IBV M protein involved in budding and maturation, thereby suggesting that its antiviral effects occur *via* the engagement of multiple targets.

Antifungal activities have been reported for the knottin peptides Mj-AMP1 from *M. jalapa* and PAFP-S from the pokeweed *Phytolacca americana* (141, 142). MJ-AMP-1 and Mj-AMP2 from *M. jalapa* also inhibit Gram-positive bacteria but are ineffective against Gram-negative bacteria (141). The solved structure of PAFP-S reveals the presence of an extended hydrophobic patch composed of both aromatic and aliphatic amino acids with neighboring cationic and hydrophobic residues; this amphiphilic character is considered to be the basis of its antifungal property (142). As their three conserved disulfide bonds can generate 15 different isomers and because the proper folding of CRPs is required for their activity, it is challenging to mass produce knottins in their proper configuration (143). An approach to overcome this constraint utilizes selenocysteine residues to form diselenide bonds at lower redox potentials than cysteine disulfides, which substitute for the cysteine pairs and induce cross-linking of the remaining cysteines (144, 145). Furthermore, heterologous expression systems with bacteria have also been developed to facilitate the production of knottins (146, 147).

### Snakin-like peptides

Snakins are CRPs with up to 12 cysteines, usually wound or infection induced and studied most in potato (*Solanum tuberosum*) (148, 149). Orthologs are found in several plants such as *Arabidopsis thaliana*, castor bean, common daisy, petunia, strawberry, and tomato (150, 151, 152, 153). The fruits of jujube (*Zizyphus jujuba*; Rhamnaceae) contain a cationic antimicrobial peptide called Snakin-Z, with activity against *S. aureus* and well tolerated by blood cells (90). Snakins produced in heterologous systems, such as *E. coli*, the yeast *Pichia pastoris*, and baculovirus-infected insect cells (154), retain their antibacterial and antifungal activities (155, 156, 157, 158). Heterologously produced Snakin-2 from *Solanum lycopersicum* inhibits *F. solani* (157) and is also reported to be active against *Bacillus subtilis, E. coli*, and *Saccharomyces cerevisiae* (159). The antimicrobial activity of snakins is suggested to be from an unspecific pore formation mechanism that leads to cell aggregation (159).

Both native and recombinant forms of the snakin-like peptide, PdSN1, isolated from the South American tree *Peltophorum dubium* (Fabaceae) and subsequently produced heterologously in *E. coli*, displayed antifungal properties against *A. niger* and *C. albicans* (160). The structural analysis showed that PdSN1 possesses a helix–turn–helix motif, which is stable under varying combinations of disulfide bonding, including when all the 12 cysteines are reduced. This suggests that the disulfide bonding is dispensable for its antimicrobial activity (160). In addition to the membrane disruption, the helix–turn–helix motif is often found in DNA-binding proteins, and combined with its positive electrostatic potential, PdSN1 may bind to DNA and interfere with microbial gene expression (160). The phenomenon is proposed as the structural basis for the mode of action of defensins as a whole (117).

### α-Hairpinin-like peptides

Members of the hairpinin family contain the unique (XnC1X3C2XnC3X3C4Xn) motif forming a characteristic helix–loop–helix structure, known as the α-hairpin (161, 162, 163). The α-hairpinin EcAMP1 from kernels of barnyard grass, *Echinochloa crusgalli*, is active against several fungal and bacterial genera. Of interest, all *Fusarium* species tested showed substantial sensitivity to EcAMP1 (91) through induced apoptosis (164). On the other hand, Sm-AMP-X, from chickweed (*Stellaria media*) seed is active against *A. niger* but is not effective on *Fusarium* spp. (163), opening up possibilities for narrow-spectrum antifungal development of the hairpinins. Furthermore, the structural elements important for the activity of EcAMP1 are two α-helices and a small cluster of positively charged amino acids, which together interact with negatively charged fungal cell wall carbohydrates, as well as fungal cell membrane lipids like sphingolipids or ergosterols (165). The hairpinin family has diverse biological activity and includes members with trypsin-inhibiting (166) and ribosome-inactivating properties (167). Hairpinins with ribosome-binding activity are of interest for the development of antiviral treatments. For instance, Luffin P1 from sponge gourd (*Luffa cylindrica*) inhibits the replication and transportation of HIV (92).

### Hevein-like peptides

Hevein is a chitin-binding antifungal protein from the rubber tree *Hevea brasiliensis* (168). The active portion of the protein is a shorter peptide 43 amino acids long and generated following co- and posttranslational processing (169). Peptides with sequences similar to that of hevein have been identified in a wide range of plants (170). The species *Eucommia ulmoides* Oliv (Eucommiaceae family) is used in Chinese herbal medicine and produces hevein-like peptides (HLPs) with activity against *F. oxysporum* and *F. solani* (171). Two mechanisms have been uncovered for the antifungal activity of HLPs; interference with chitin assembly (172) and disruption of the fungal cell membrane by ionic interactions (173, 174). The molecules known as bleogens from *Pereskia bleo* (Cactaceae) are HLPs; one of these, pB1, contains the cystine-knot disulfide motif, β-sheets, and a motif containing four loops. It is antifungal with low micromolar minimum inhibitory concentrations against *C. albicans* and *C. tropicalis*, while showing no cytotoxicity toward mammalian cells (93). The seeds of wheat *Triticum kiharae* produces a 10-Cys peptide, which inhibits *F. oxysporum* and *F. solani* through chitinase activity (175). The medicinal plants of the Ginseng group (genus *Panax*) contain a novel class of HLPs, peptides rich in cysteine and glycine, called ginsentides, containing a pseudocyclic structure that confers heat and proteolytic degradation resistance (176). Recently, the gymnosperm *Ginkgo biloba* was shown to produce proline-rich acid-stable HLPs called gingkotides, which inhibit *A. niger* and *F. oxysporum,* and bioinformatic analysis suggests that gingkotide-like HLPs are ubiquitous throughout gymnosperms (177).

### Other antimicrobial peptides

Among the best understood CRPs outside of the categories discussed above are the cationic 6- to 8-Cys peptides ToAMP1, ToAMP2, and ToAMP3, produced by the flowers of the common dandelion *Taraxacum officinale* (178). All three peptides have antifungal activities against *A. niger* and antibacterial activities against *B. subtilis*, whereas ToAMP3 also inhibits *F. oxysporum* (178). The ToAMPs display unusual spacing between the cysteines and form a separate class of CRPs found so far only in *T. officinale* (178). The seeds of the wax gourd, *Benincasa hispida,* produce a cationic peptide called hispidalin, which inhibits the fungus *A. flavus* and the bacteria *B. cereus, E. coli*, *P. aeruginosa*, *S. aureus,* and *S. enterica* at concentrations comparable with commercially available drugs (179). Active hispidalin has been heterologously produced in *P. pastoris* and shown to have protease stability and low hemolytic toxicity even at 300 μg/ml (180). Several bean species produce trypsin-resistant defensive peptides in their seeds; for example, vulgarinin produced by haricot beans (*Phaseolus vulgaris*) is fungicidal against *F. oxysporum* (181), inhibits several bacteria such as *Bacillus megaterium*, *B. subtilis, Mycobacterium phlei*, and *Proteus vulgaris,* and also inhibits the HIV reverse transcriptase (181).

## Unusual amino acids and derivatives

Hundreds of amino acids not involved in peptide synthesis are produced in the plant kingdom. Instead, these amino acids are used for defensive functions such as deterring herbivores, pests, and pathogens or for allelopathy (182). Although many of these nonproteinaceous amino acids (NPAAs) display toxicity to animals, they show promise for their anticancer or neuroprotective effects and several studies have explored their antimicrobial properties against human pathogens (183).

**Figure 1:**
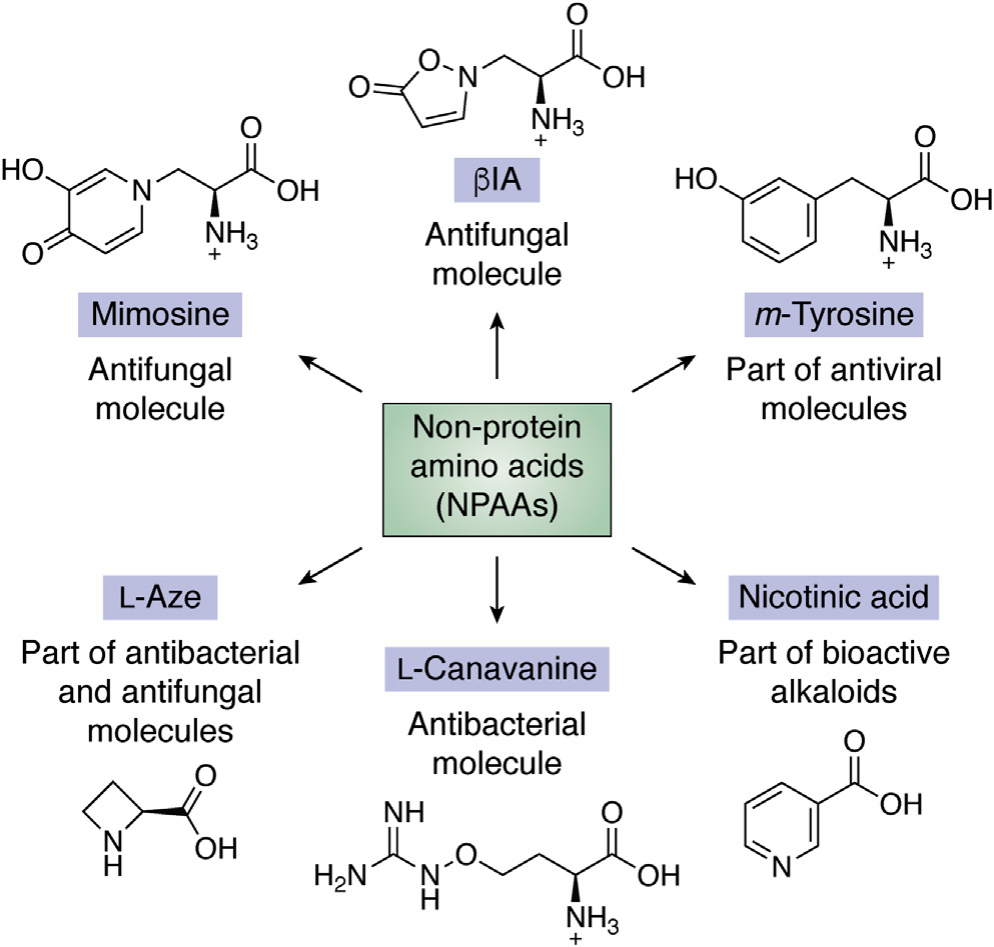
**Nonprotein amino acids (NPAAs) with anti-infective properties.** Mimosine, leucenol or β-[N-(3-hydroxy-4-pyridone)]-aminopropionic acid (antifungal), β-(3-isoxazolin-5-on-2-yl)-alanine or βIA (antifungal), *m*-Tyrosine (part of antiviral molecules), nicotinic acid (part of bioactive alkaloids), l-canavanine (antibacterial), and azetidine-2-carboxylic acid, l-Aze, or A2C (part of antibacterial and antifungal molecules).

Mimosine (also known as leucenol or β-[N-(3-hydroxy-4-pyridone)]-aminopropionic acid; Figure 1), produced by the seeds, leaves, and roots of several Fabaceae (184, 185, 186), has potent activity against the dermatophytic fungi *Trichophyton rubrum* and *Trichophytum tonsurans* (187). Pea (*P. sativum*) seedlings produce β-(3-isoxazolin-5-on-2-yl)-alanine (βIA; Fig. 1) (188), which has broad-spectrum antifungal activity, including against *S. cerevisiae* (188, 189). Aside from their biological activities as free amino acids, some NPAAs are incorporated into larger molecules. *m*-Tyrosine (Fig. 1) is produced by many grasses as an herbicide (190), and macrocycles containing *m*-Tyrosine have been developed as viral protease inhibitors (191). Ornithine, nicotinic acid (Fig. 1), anthranilic acid, and some β-hydroxy amino acids are also precursors for several classes of alkaloid compounds (192, 193).

l-Canavanine (Fig. 1), produced by leguminous plants such as *Medicago sativa*, interferes with quorum sensing in root-colonizing soil bacteria (194), likely through inhibiting bacterial arginine deiminase. Arginine deiminase is absent in humans, and it provides beneficial traits to bacterial pathogens making it an attractive antibacterial and antiparasitic drug target (195, 196). Azetidine-2-carboxylic acid or A2C (Fig. 1) contains an unusual four-membered heterocycle, produced by lily of the valley (*Convallaria majalis*) (197) and beet (*Beta vulgaris*) (198). A2C is an analog of both proline and alanine and activated by both human prolyl-and alanyl-tRNA synthetases, leading to misincorporation in proteins and protein toxicity (199). However, owing to its toxicity, free A2C is generally avoided and instead the azetidinone moiety is preferred in drug development. Synthetic A2C derivatives are effective against bacterial and fungal pathogens (200). Of interest, azetidinone incorporated into semisynthetic penicillins result in low cytotoxicity and improved efficacy against *Staphylococcus* sp. (201).

## Alkaloids

Alkaloids are widely distributed in crop species and in medicinal plants employed over several millennia (202). Alkaloids are undeniably the best understood plant secondary metabolites and include atropine, caffeine, codeine, morphine, quinine, strychnine, theobromine, and xanthine. The alkaloid class covers many defense compounds and comprise a paraphyletic chemical group with regards to their biosynthesis where small molecules with one or more basic nitrogen atoms are considered alkaloids (203). These include compounds incorporating nitrogen from amino acids into heterocyclic rings (true alkaloids) or outside of the heterocyclic ring (protoalkaloids) (204). Over 27,000 alkaloids are currently listed in the Dictionary of Natural Products (205), and the number is continuously growing. The true alkaloids are derived primarily from the aromatic amino acids, namely, phenylalanine, tyrosine, and tryptophan, whereas NPAAs can also contribute to their biosynthesis. True alkaloids are classified based on the heterocyclic structure (192) and more than 2500 sub-ring skeleton types have been detected in the KNApSAcK Core Database of 12,000 alkaloids (206). The enormous potential of alkaloids as drug leads is far from exhausted and a variety of pharmacological effects continues to be reported and reviewed (207, 208, 209, 210, 211, 212). Owing to the extensive diversity and immense number of alkaloids, we will only discuss selected examples of antibacterial, antifungal, antiviral, and antiparasitic molecules, with an emphasis on those reported in the last 10 years. A brief summary of the alkaloids with the most promising *in vivo* studies is shown in Table 2.

**Table 2:** A summary of the *in vivo* antimicrobial activity of promising alkaloids and organosulfur compounds

Compound (class, plant source)	Mechanism of action	Target pathogen/infection (reference)	Relevance
α-Chaconine (steroidal glycoalkaloid, Solanaceae)	Suppresses 70% of the parasites over 4 days	*Plasmodium yoelli* (213)	Pervasive drug resistance of malarial parasites
Lycorine (phenylethylamine alkaloid, wild daffodil)	Inhibits RNA-dependent RNA polymerase, reduces viral load	Zika virus (214)	Approved vaccines/specific antivirals not available
l-Ephedrine, d-pseudoephedrine (phenylethylamine alkaloid, *Ephedra* spp.)	Mitigate lung injury, decrease viral load and serum interleukin IL-1β, reduce levels of inflammatory factors, increase serum interleukin 10 and interferon γ	Influenza A virus (215)	Improves host immune defenses post infection
Berberine (isoquinoline alkaloid, Berberidaceae)	Globally reduces viral activation of major mitogen-activated protein kinase pathways, reduces viral titer and inflammatory symptoms	Chikungunya virus (216)	Attacks multiple targets and suppresses host inflammation
MFM501 (synthetic derivative of pyrrolidine alkaloid from *Codonopsis clematidea*)	Bacteriostatic against over 40 clinical strains, targets the bacterial membrane	Methicillin-resistant *S. aureus* (252, 253)	Clinical strains suppressed with no toxicity
Voacamine (indole alkaloid, *Tabernaemontana coronaria*)	Kills parasites by poisoning topoisomerase 1B; does not inhibit human topoisomerases I and II	*L. donovani, L. amazonensis, T. cruzi* (217)	First molecule active against *L. donovani* strains resistant to sodium antimony gluconate, amphotericin B, and miltefosine
Allicin (organosulfur, garlic)	S-allylmercaptyl addition to bacterial cysteine sulfides, depletion of glutathione pools, induction of heat stress response; inhibits diesterases and oxidoreductases, disrupts plasma and endomembranes, promotes apoptosis and cell cycle arrest in parasites (reduces load, kills trophozoites)	Lung pathogenic bacteria, *Giardia duodenalis* (279, 291)	Only inhalable antibiotic to clear lung infection; resistance to anti-giardial metronidazole rising, poor vaccine availability

### Pseudoalkaloids

In these compounds, the carbon skeletons are not derived from amino acids and the nitrogen is usually incorporated by a transamination reaction. Pseudoalkaloids include the steroidal alkaloids of the Solanaceae family and glycoalkaloids. The identification of biosynthetic genes for steroidal alkaloids (218, 219) and the optimization of yeast platforms (220) allow for the customization and biotechnological production of these molecules.

### Tomato alkaloids

**Figure 2:**
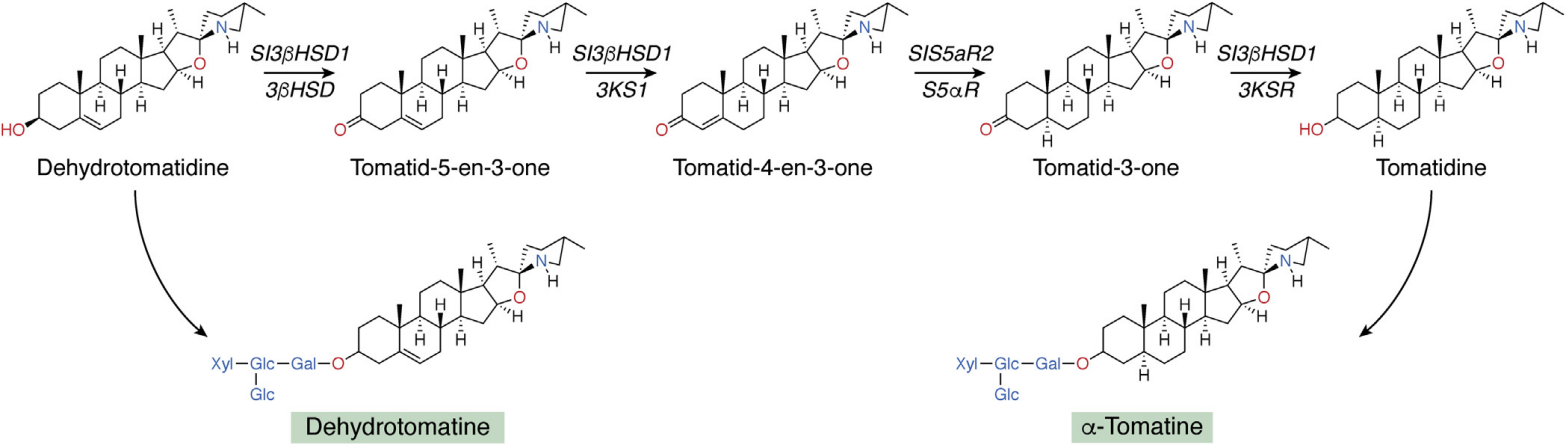
**The biosynthetic pathway of the tomato alkaloids based on Akiyama *et al*.** (221)**.** The nitrogen incorporation occurs in the earlier phase of the biosynthesis from cholesterol (222). The genes names in tomato are shown as *yellow* entries, while the *blue* entries are the enzyme activities. 3βHSD, 3β-hydroxysteroid dehydrogenase; 3KSI, 3-ketosteroid isomerase; S5αR, steroid 5α-reductase; 3KSR, 3-ketosteroid reductase.

The tomato plant, *S. lycopersicum* L. produces the cholesterol-derived steroidal alkaloids tomatine and tomatidine. A summary of their biosynthesis from the precursor dehydrotomatidine *via* enzymatic dehydrogenation, isomerization, and successive reductions is shown in Figure 2, which is based on (221). Tomatidine exerts a selective and potent inhibitory effect against small-colony variants of *S. aureus* that cause opportunistic infections in patients with cystic fibrosis (223). Mutant and pharmacological analysis identified electron transport dysfunction as the major mechanism for the effect of tomatidine, which holds promise as a novel antibiotic lead against persistent forms of chronic *S. aureus* infections. Tomatidine also has potent fungistatic activity against *Candida* spp. with low toxicity to human cells (224). Transcriptional and biochemical analysis led to the finding that tomatidine inhibits sterol methyltransferase and reductases. It is remarkable that tomatidine also shows antiviral activity *in vitro* against the chikungunya virus (CHIKV), for which vaccines and antiviral compounds are not currently available (225). Tomatidine inhibits viral particle production after the entry of the virus into mammalian cells, and its activity persisted for 24 h after infection, suggesting that it blocks multiple rounds of viral replication.

### Other Solanaceae alkaloids

The surfactant-like saponins are widely distributed in over 100 plant families and consist of terpenoid or steroidal glycoalkaloid compounds (226, 227). Solanaceous plants produce steroidal alkaloids possessing broad spectrum activity against multiple groups of pathogens. The commonly occurring α-chaconine and α-solanine show strong antifungal properties (228). The glycoalkaloids chaconine, solanine, solamargine, and tomatine were tested for antimalarial activity against *Plasmodium yoelli* in murine models (213), with the best antimalarial activity shown by chaconine. The replacement of the sugar moiety reduced the activity of the glycoalkaloids, suggesting that carbohydrate interactions are required for their antimalarial properties (213). Furthermore, sulfation of the 6-OH group led to loss of activity, demonstrating that this group is also critical for the pharmacological effects (213). Oral doses of most Solanaceae alkaloids of 3 to 5 mg/kg body weight in humans are toxic. However, mice can tolerate injections of chaconine at 7.5 mg/kg body weight with an ED50 (effective dose to reach 50% response in 50% of the subjects) of about 4.5 mg/kg body weight and a therapeutic index of 9 against *P. yoelli* infections (213). In the light of pervasive resistance to antimalarials, the Solanaceae alkaloids hold promise for further development.

### Protoalkaloids

Protoalkaloids contain the amino acid–derived nitrogen outside of the heterocyclic ring. The two major families in this category are the terpenoid-containing indole alkaloids and the phenylethylamine alkaloids.

### Terpenoid indole alkaloids

These are commonly found in plants of the dogbane (Apocynaceae) family, which includes *Ervatamia chinensis*, *Voacanga africana*, and the blackboard tree (*A. scholaris*). Indole alkaloids of *E. chinensis* possess antibacterial and antifungal activities (229). The bioactive compounds erchinine A and B contain a unique 1,4-diazepine structure joined to an oxazolidine and showed activities against the fungus *T. rubrum* comparable with the standard antifungal drug griseofulvin, whereas the inhibitory effect on the bacterium *B. subtilis* was comparable with that of the antibiotic cefotaxime (229). Although *T. rubrum* is generally non-life threatening, chronic *T. rubrum* infections facilitate secondary fungal infections, which can become lethal when systemic (230, 231). Although the mechanisms of action are not yet understood, erchinine A and B are promising for the development of novel antifungal leads.

*Aspidosperma olivaceum* is a Brazilian medicinal plant, which contains several antimalarial compounds, with aspidoscarpine displaying promising activity and selectivity against the bloodstream forms of chloroquine-resistant *Plasmodium falciparum* and *T. brucei* (232)*. Buxus sempervirens* extracts are used as an antimalarial, and its pharmacological effect is best explained by the presence of the cycloartane alkaloid *O*-tigloylcyclovirobuxeine-B, which shows selectivity against *P. falciparum* at low concentrations (233). Of importance, it was shown that cytotoxic and antimalarial/antitrypanosomal activities are due to other compounds in the extracts, and these compounds could be readily separated (233). The antibacterial and antiparasitic mechanism of this compound class is unclear, but earlier work suggests that they may inhibit DNA topoisomerase or intercalate DNA (234, 235).

### Phenylethylamine alkaloids

**Figure 3:**
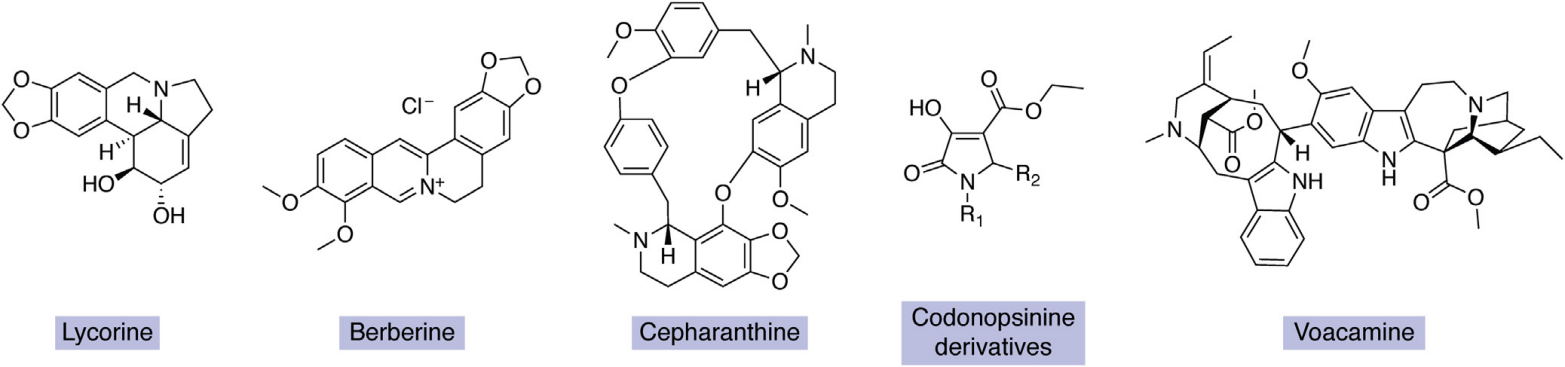
**Selected alkaloids, which have been utilized in *in vivo* studies: lycorine, berberine, cepharanthine, codonopsinine derivatives and voacamine**.

Lycorine (Fig. 3) is a benzyl phenethylamine alkaloid that was first isolated from the wild daffodil (*Narcissus pseudonarcissus*). Cedrón *et al*. synthesized and evaluated 27 derivatives of lycorine and found that the hydroxylation/esterification of the C1 or C2 positions and the presence of the double bond between C2 and C3 positions were essential for its antimalarial activity (236). Lycorine also inhibits flaviviruses such as West Nile virus (WNV), dengue virus (DENV), and yellow fever virus; however, a single amino acid substitution in the WNV 2K peptide was sufficient to confer lycorine resistance (237). In mice models, lycorine also possesses antiviral activity against the Zika virus (ZIKV) and inhibits RNA-dependent RNA polymerase (214) and, as a consequence, decreases the viral load. This is an important development since currently no vaccine or specific antiviral treatment is approved for ZIKV.

Substituted phenylethylamines are among the bioactive substances produced by *Ephedra* spp. (Ephedraceae) and commonly used as bronchodilators. The major *Ephedra* alkaloids are l-ephedrine, d-pseudoephedrine, and l-methylephedrine, which have antiviral effects on influenza A virus (IAV) *in vitro*, through inhibition of viral replication and modification of the inflammatory response (215). Of more importance, studies in mice showed that, after infection, l-ephedrine and d-pseudoephedrine mitigated lung injury, decreased the viral load and serum interleukin 1β, reduced transcription and translation of several inflammatory factors, and also increased the level of serum interleukin 10 and interferon γ (215) expression. Apart from their *in vitro* activity, the ability of *Ephedra* alkaloids to ameliorate host inflammation and induce antiviral host defenses against IAV make them promising candidates for clinical application.

### True alkaloids

These contain one or more basic nitrogen elements and carbon skeletons derived from preotegenic and nonproteogenic amino acids. Selected classes are discussed here.

### Cyclopeptide alkaloids

These are compounds with a 13-, 14-, or 15-membered macrocyclic ring system with 4 to 5 moieties comprising an amino acid, a β-hydroxy-amino acid, a hydroxystyrylamine moiety, and further substituents on the rings (238, 239). They are most widely distributed in the Acanthaceae, Malvaceae, Phyllanthaceae, Rhamnaceae, and Rubiaceae families; their structural diversity, pharmacological activities, syntheses, and antimalarial activity have been recently reviewed (193, 240). Fourteen-membered cyclopeptide alkaloids from the Brazilian medicinal plant *Discaria americana* (Rhamnaceae) showed antibacterial activity against *E. coli, Enterobacter aerogenes, Enterobacter faecium*, and *S. enterica* (241). Mauritine-M and nummularine-H showed satisfying activity against *Mycobacterium tuberculosis;* the latter had an effect comparable with that of the frontline antibiotic isoniazid and was also able to target MDR strains (242).

*Hymenocardia acida* produces the antimalarial hymenocardine and other cyclopeptide alkaloids endowed with moderate activity, good selectivity, and low human cytotoxicity, and these could be employed as lead compounds for further optimization (243). A 2017 study of several cyclopeptide alkaloids revealed that their antimalarial activity is increased if their macrocycle is 13-membered and methoxylated at position 2 of the styrylamine. The effect of modification of the β-hydroxy proline and aliphatic amino acids in the macrocycle remain unclear (240).

### Isoquinoline alkaloids

These are found in several plant families such as Berberidaceae, Fumariaceae, Lauraceae, Menispermaceae, Papaveraceae, and Ranunculaceae and often possess antibacterial activity. Recently, enantioselective synthetic methods were developed for the reduced isoquinoline alkaloids, norglaucine, nordicentrine, and dicentrine, which showed promising activity against the parasites *Leishmania* *infantum* and *Trypanosoma cruzi* (244). The anti-*T. cruzi* alkaloid dicentrinone from *Ocotea puberula* (Lauraceae) causes disruption of parasite cell membranes *via* multiple mechanisms (245). From over 140 alkaloids tested, the most effective antimalarial was jozimine A2 from *Ancistrocladus* spp., which inhibited *P. falciparum* NF54 in the low-nanomolar range. Jozimine A2 was also nontoxic to mammalian cells and highly selective for *P. falciparum* as opposed to other parasites (246), making it an excellent lead molecule for further antimalarial research.

Berberine (Fig. 3) is a well-known benzylisoquinone alkaloid from the family Berberidaceae and inhibited the CHIKV in various cell lines (216). Furthermore, berberine is effective against several CHIKV strains without any direct effect on viral replication and significantly decreases the viral activation of the major mitogen-activated protein kinase (MAPK) signaling pathways. However, unlike specific kinase inhibitors, berberine decreased the viral activation of all major MAPK pathways, resulting in a marked reduction of the viral titer. Finally, *in vivo* mice models treated with berberine showed strong efficacy with an appreciable reduction of the Chikungunya-associated inflammatory symptoms (216).

Cepharanthine (Fig. 3) is a bisbenzylisoquinoline alkaloid from the Asian medicinal plant *Stephania cepharantha* (Menispermaceae) and approved for clinical use in Japan. It has an established safety record and is employed for its antiparasitic and antiviral properties, as well as several health benefits (247). Several mechanisms explain its antimicrobial activities including interference with efflux pumps, membrane rigidification, modulation of the AMP-activated protein kinase, and impacting the nuclear factor kappa-light-chain (NF-κB) signaling pathways (248). It suppresses several processes critical for both viral replication and the host inflammatory response, such as activation of nuclear factor NF-κB, lipid peroxidation, cyclooxygenase expression, and nitric oxide (NO) and cytokine production (249). Among the over 2400 clinically approved drugs screened in a repurposing effort for the current COVID-19 pandemic, cepharanthine was the most potent and capable of inhibiting both the entry and replication of SARS-CoV-2 and similar viruses providing solid rationale for its use in antiviral development (249). However, it has so far not been economically synthesized (248).

### Pyrrolidine alkaloids

Plants of the Amaryllidaceae family commonly produce pyrrolidines. The Asian bellflower (*Codonopsis clematidea*; Campanulaceae) contains unusual aromatic substituted pyrrolidines with antibiotic activities including codonopsinine (250, 251). A hydroxylated codonopsinine derivative (Fig. 3), MFM501, has bacteriostatic activity against more than 40 clinical MRSA strains targeting cellular membranes and is tolerated by mice with no toxic effects (252, 253). Friar's cowl (*Arisarum vulgare*; Araceae) contains the antibacterial and antifungal compound (*R*)-bgugaine. The synthetic demethylated form, (*R*)-norbgugaine, was synthesized and shown to inhibit quorum sensing in *P. aeruginosa*, which in turn suppressed motility, biofilm formation, pyocyanin pigmentation, rhamnolipid production, and the LasA protease (254).

### Indole alkaloids

**Figure 4:**
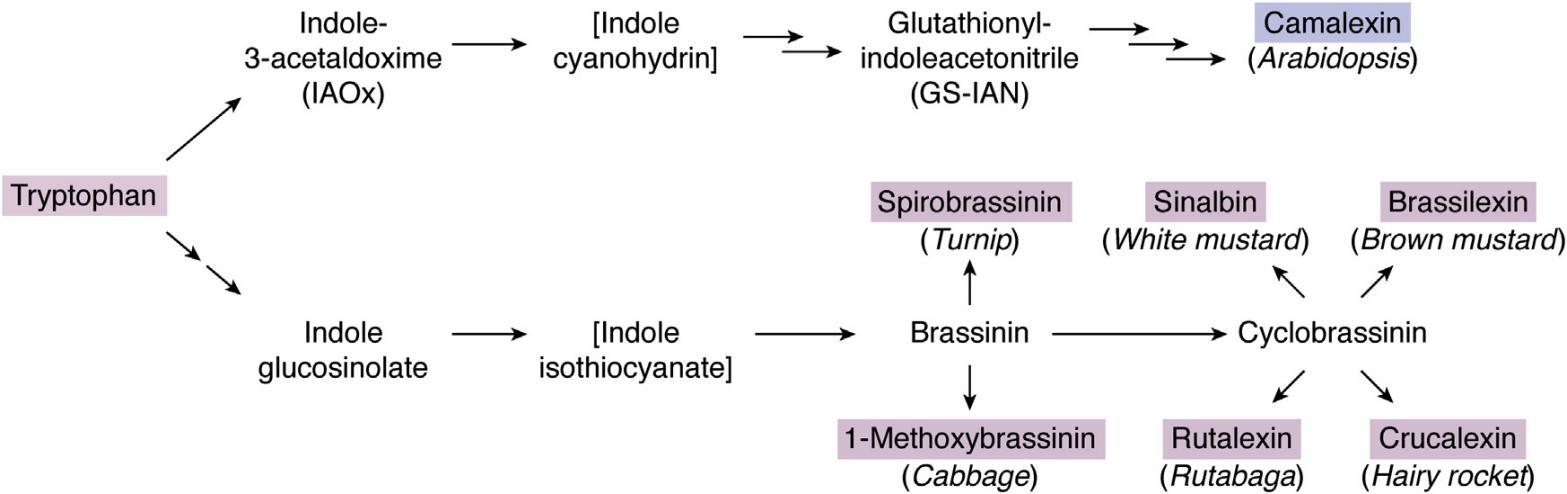
**The biosynthetic pathway of camalexin *via* indole-acetaldoxime (*top*) based on Mucha *et al*.** (261) **and that of cruciferous indoles *via* indole glucosinolate (*bottom*) based on Klein and Sattely** (262)**.** The *dashed arrows* and the *square brackets* emphasize proposed unstable intermediates. The common names of plants producing some compounds are shown italicized in parentheses.

A comprehensive survey of indole alkaloids showed that 261 new compounds of this class were discovered from plants in the Annonaceae, Apocynaceae, Loganiaceae, and Rubiaceae families since 2012 (255). The β-carboline nigritanine from the African tree *Strychnos nigritana* Baker (Loganiaceae) inhibits clinical *S. aureus* strains, with no toxicity to mammalian cells, and structure–activity studies showed that dimerization improves its antibacterial activity (256). Voacamine (Fig. 3) obtained from *Tabernaemontana coronaria* is a broad-spectrum antiprotozoal active against *Leishmania donovani*, *Leishmania amazonensis*, and *T. cruzi*, and with especially high specificity for the *L. donovani* topoisomerase 1B. It is the first molecule demonstrated in mouse models to be active against *L. donovani* strains that are recalcitrant to standard drugs, while having no inhibitory effect on the human topoisomerases I and II (217). The blackboard tree (*A. scholaris*) contains the unusual antiviral 17-nor-excelsinidine, shown to inhibit the herpes simplex virus and adenovirus in transfected cells and to be more effective than the antiviral drug acyclovir (257). Indole alkaloids contain the unique spirooxindole 3-dimensional structure and are produced by many medicinal plants. Their antiviral properties (215) make them enticing lead molecules and has spurred research to understand the effects of chemical modifications (258). Of the synthesized compounds, spiropyrazolopyridones were identified as potent DENV replication inhibitors, and lead optimization produced an orally bioavailable preclinical spiropyrazolopyridone effective in mouse models. This compound showed an impressive 80-fold reduction of viremia (259). Camalexin is an archetypical PA containing the indole-thiazole structure produced by Brassicaceae, including the model plant *A. thaliana*, in response to fungal or bacterial pathogens (260). The biosynthesis of camalexin from tryptophan *via* indole-3-acetaldoxime is shown in Figure 4 based on Mucha *et al*. (261). The indole-containing PAs of crucifers are biosynthesized from tryptophan *via* indole glucosinolate (Fig. 4) (262). The cruciferous PAs have antifungal effects, but resistance frequently occurs in plant pathogenic fungi (70, 263, 264). Nonetheless, proteomic studies have identified the heat shock protein HSP90 as the antifungal target of brassinin-type compounds (265) and fungal-specific brassinin analogs can exploit the structural differences between the *C. albicans* and human HSP90 enzymes (266). The recent elucidation of the biosynthetic pathways of indole- and sulfur-containing cruciferous PAs offers promise in developing novel analogs *via* metabolic engineering efforts (262). Camalexin derivatives are predominantly used in anticancer drug development, whereas compounds containing synthetic elaboration of its core structure confer selective antibacterial properties against Gram-negative bacteria (267). This is important since the majority of natural and synthesized antibacterial agents cannot pass the extra outer membrane enveloping Gram-negative bacteria, a structure hypothesized to have evolved for defense against the small molecule arsenal produced by competing bacteria (256, 268).

### Quinolizidine alkaloids

These are known largely from leguminous plants of the Fabaceae family, especially *Sophora* spp., and include several antiviral molecules.

Quinolizidine alkaloids have been investigated extensively for anti-influenza activities (269). Several plants from the genera *Laburnum* and *Cytisus* contain (-)-cytisine, whose synthetic derivatives have been evaluated against the IAV H1N1 strain and the human parainfluenza virus type 3, whereby good selectivity and negligible toxicity were observed (270). *In silico* models suggest that the 9-carboxamides of methylcytisine bind the active site of IAV neuraminidase, whereas (-)-cytisine and 9,11-dibromocytisine were shown to block human parainfluenza virus type 3 reproduction, with predicted selectivity indices of 58 and 95, respectively (270). Dang *et al*. identified *Sophora* alkaloids, including dihydroaloperine, which in cell cultures inhibits an H1N1 strain of IAV resistant to two frontline antivirals, oseltamivir and amantadine (271). The mechanism of action was shown to be the inhibition of the IAV nucleoprotein; additional targets may include a viral protein involved in the different stages of replication (271).

The quinozolidine alkaloid sparteine contains a bicyclic bispidine core, which is considered a privileged scaffold (272). Bispidine was used to generate several synthetic amino acid conjugates that were tested in cell cultures against Japanese encephalitis virus (JEV). A bispidine-tryptophan conjugate inhibited JEV infection by more than 100-fold, likely by suppressing RNA replication (273). The pharmacological effect of the conjugates has been attributed to the rigidity conferred by the bicyclic bispidine and the presence of linked amino acids, which mimic protein secondary structures disrupting protein–protein interactions. These molecules are especially promising as there are no antivirals specifically approved for JEV, and in endemic areas, pediatric vaccination coverage is poor, and thus making the development of small molecule leads an urgent need.

## Organosulfur compounds

**Figure 5:**
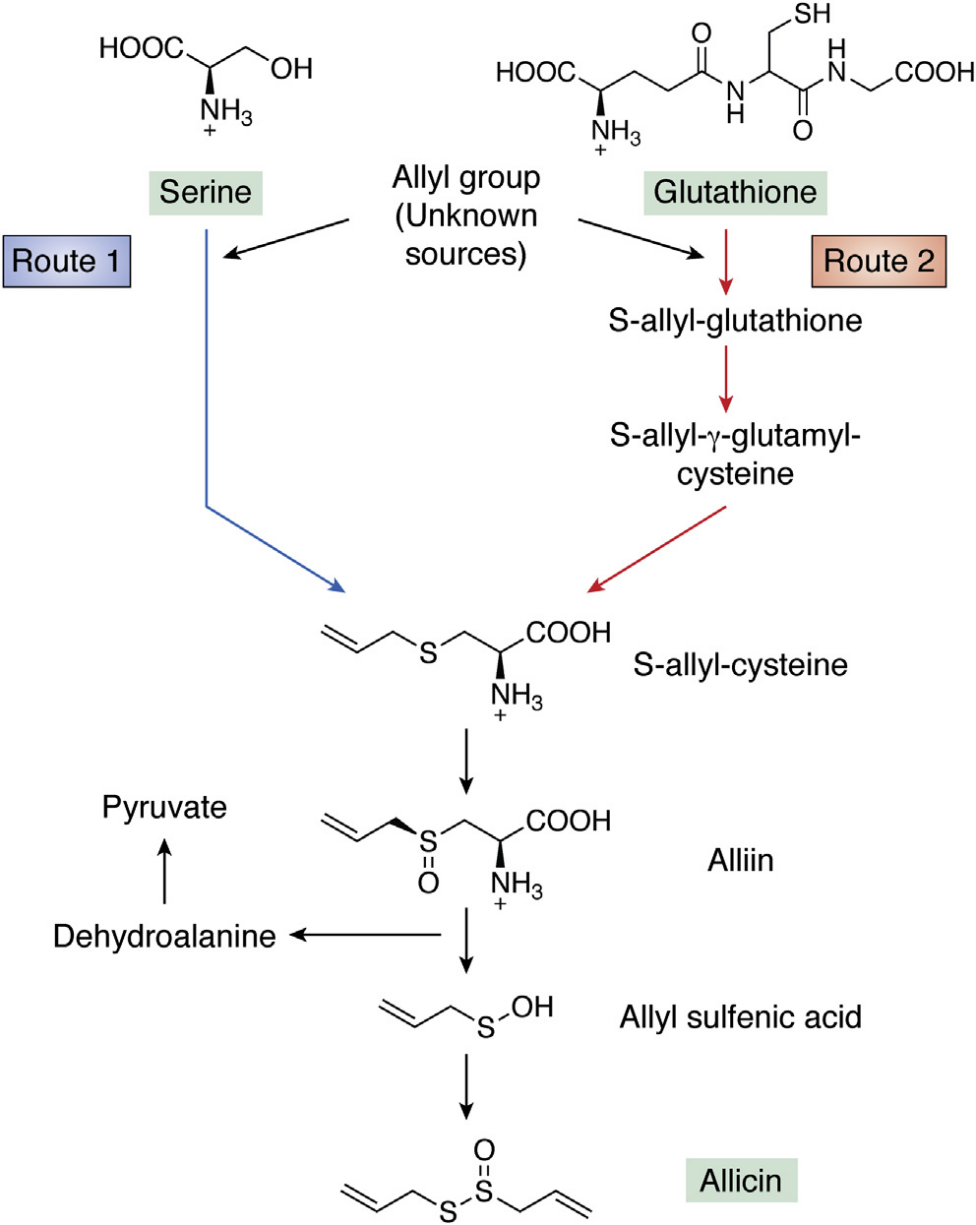
**Two possible biosynthetic pathways (Routes 1 and 2) of the linear sulfurous compound allicin from the precursors serine and glutathione based on** (276)**.** The immediate precursor of alliin is S-allyl cysteine, which may derive from either serine or glutathione. 14C-Labeled serine feeding experiments led to the formation of 14C-labeled S-allyl-cysteine. However, S-allyl-glutathione and S-allyl-γ-glutamyl-cysteine have been detected in other experiments. The source of the allyl group is unknown for both Routes 1 and 2.

Garlic (*Allium sativum*) produces the amino acid–derived organosulfur defense compounds allicin, ajoene, alliin, diallyl disulfide, and diallyl sulfide. The most famous of these is allicin, which is widely distributed in the Alliaceae family and was first characterized in the 1940s as an antibacterial (274, 275). The uses of allicin have been summarized recently (276) and mentioned in Table 2. Allicin biosynthesis based on (276) is shown in Figure 5; the serine or glutathione precursors form an S-allyl adduct or S-allyl glutathione, respectively, and are metabolized further into additional defense compounds. The source of the allyl group is yet unknown. Earlier studies showed that subcutaneous administration of garlic extracts led to rapid clearance of pulmonary *P. aeruginosa* infections in mice (277), but the active compound was not identified. In a later randomized controlled clinical trial with patients with cystic fibrosis, allicin was administered orally (instead of garlic extracts) and failed to achieve pharmacologically effective concentrations owing to interference from glutathione dissolved in the bodily fluids (278). However, in contrast to oral administration, allicin vapors kill human lung pathogens including MDR strains in cell cultures as well as rat lung tissues without causing cell/tissue damage (279). In addition, there is some evidence that thiol compounds cause the breakdown of mucin monomers to polymers, leading to mucus clearance (280). Furthermore, the cytotoxicity of allicin in both human and murine cells is mitigated by glutathione without reducing its antimicrobial activity.

Allicin also synergizes with silver nanoparticles to control cutaneous MRSA (281) and suppresses quorum sensing in the Gram-negative pathogens, *P. aerugin*o*sa* (282) and *Proteus mirabilis* (283). Its antibacterial mechanism was recently elucidated as the S-allylmercaptyl addition to bacterial cysteine sulfide residues, causing thiol stress in bacteria *via* the depletion of glutathione pools and the induction of the heat stress response (284). Garlic produces hydrophobic unsaturated sulfur compounds used as starting points to generate synthetic quorum sensing inhibitors (285). The antitubercular activity of ajoene in macrophages was derived from c-Jun N-terminal kinase (JNK) activation, ROS production and accumulation, resulting in autophagy killing *M. tuberculosis* (286). This is a significant finding since reservoirs of *M. tuberculosis* inside macrophages evade the immune system and allow long-term persistence (287). Ajoene also suppresses biofilm formation *via* quorum sensing in *P. aeruginosa* and *S. aureus* by interfering with small regulatory RNAs (288, 289). Owing to the increasing threat of AMR to agents inhibiting bacterial protein, DNA, and cell wall biosynthesis, bacterial small RNAs and their inhibitory factors are considered promising targets for antibiotic development (290).

Thioallyl compounds from garlic, including allicin, inhibited the parasitic infection giardiasis or “beaver fever,” a diarrheal disease caused by *Giardia duodenalis* (291). The mode of action is through allicin-mediated thiol stress inhibition of diesterase and oxidoreductase activities, the disruption of plasma and endomembranes, the promotion of apoptosis, and cell cycle arrest (291). Although treatments are available for giardiasis, resistance to the frontline drug metronidazole is a concern and vaccines are not widely available (292). Trials with infected gerbils showed that allicin reduced the parasite load and killed the actively feeding parasite stage (trophozoites) (291). Thus, the multifactorial anti-giardial action and *in vivo* efficacy of the widely available phytochemical allicin is encouraging for further therapeutic development.

Allicin has antiviral activities against Kaposi sarcoma–associated herpesvirus, which triggers the development of Kaposi sarcoma, a skin cancer common among immunocompromised patients that can lead to chronic or acute infections (293). Current frontline antivirals acyclovir, ganciclovir, and famciclovir target only the lytic cycle of KHSV (294); however, no current treatments exist for infections caused by the latent stage. Allicin, which also inhibits the latent phase, is an attractive lead for single/combined antiviral therapy (293).

The garlic sulfur compounds diallyl disulfide, diallyl sulfide, and alliin downregulated the oxidative stress response during infection by DENV and reduced inflammation in cell lines, offering a potential way to ameliorate the progression of severe DENV symptoms (295). *In silico* modeling of the H1N1 strain of IAV with the well-known antiviral target, neuraminidase, suggested that allicin and ajoene may suppress H1N1 infection by inhibiting this critical enzyme (296). In the docking models, these two compounds target different amino acid residues of the H1N1 neuraminidase; allicin interacts with Lys150 and Gln136 and ajoene with Arg152 (296). More detailed analysis suggests that they block the sialic acid site of the enzyme and thus prevent it from cleaving sialic acid from the glycans of the host cell to initiate viral attack.

## Major aromatic amino acid–derived compounds—the phenylpropanoid pathway

**Figure 6:**
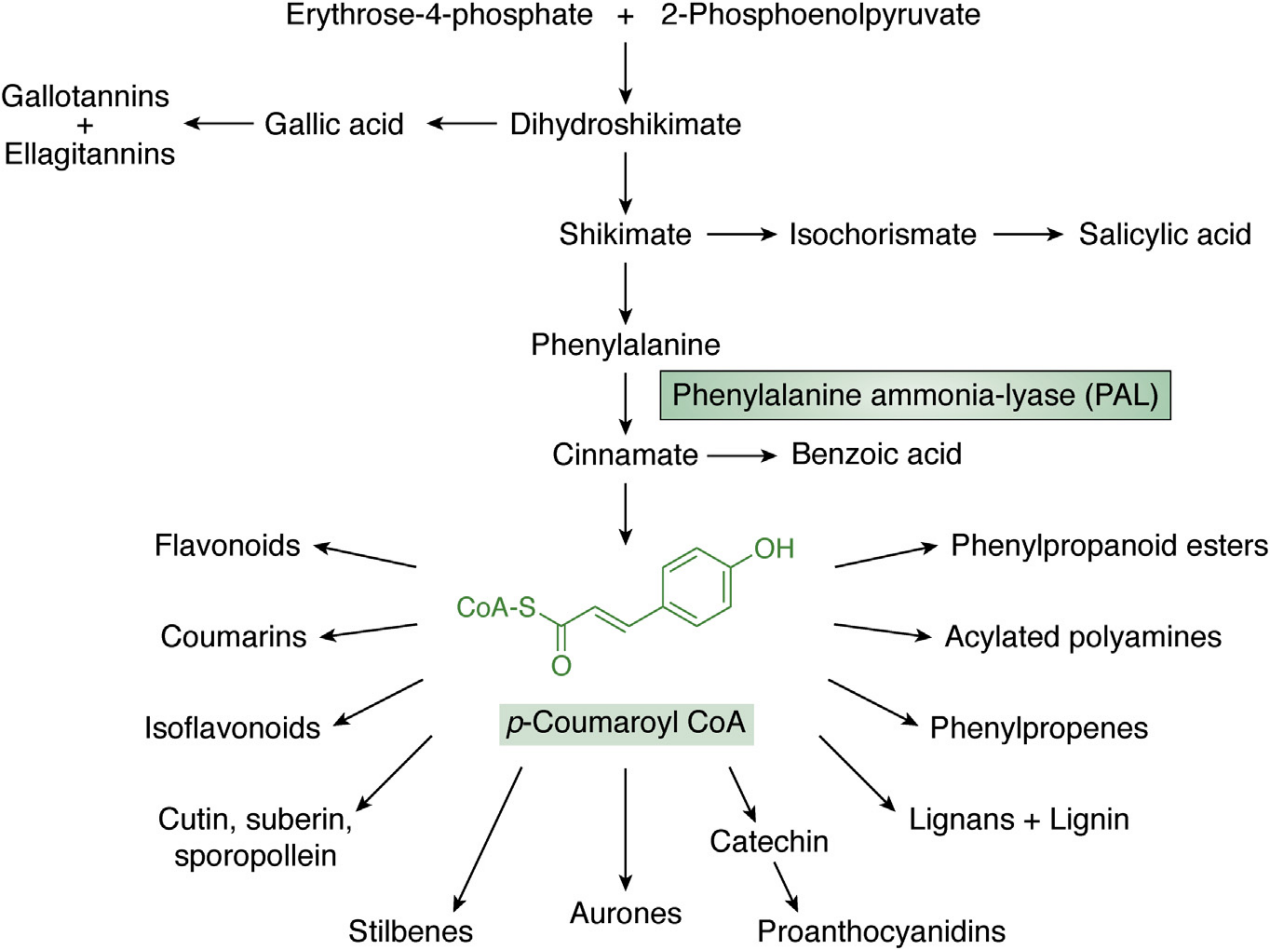
**The phenylpropanoid pathway, which leads to a variety of plant defensive compounds starting from phenylalanine *via* the central intermediate *p*-coumaroyl-CoA highlighting one of the key enzymes, phenylalanine ammonia lyase (PAL)** (298).

The phenylpropanoid (PP) pathway is a major route for the production of plant defense compounds (297). It involves the key biosynthetic enzyme phenylalanine ammonia lyase (Fig. 6), which converts phenylalanine to cinnamate (299). This pathway produces a variety of defense compounds *via* the central intermediate *p*-coumaroyl-CoA, such as anthocyanidines, coumarins, flavonoids, isoflavonoids, lignins, phenylpropenes, and stilbenes as well as others (Fig. 6) (300). All these classes include anti-infective molecules. Molecular docking simulations have implicated many PPs as promising antiparasitic (301) and antiviral agents (296). The shikimate pathway upstream of phenylalanine also funnels intermediates into the synthesis of other protective compounds such as tannins (Fig. 6). Compounds such as phenalenones may be produced *via* direct enzymatic conversions of cinnamate or phenylalanine (302). Type III polyketide synthases are typical of plants and mediate the synthesis of a variety of compounds such as chalcones, curcuminoids, benzophenones, biphenyls, and phenalenones from PP intermediates and constitute a link to the biosynthesis of alkaloids such as quinolones, alkylquinolones, and acridones (303). Here, we discuss selected examples of phenylalanine-derived compounds lacking nitrogen, in terms of their activities against various infectious disease agents. Although a number of them report *in vitro* results, a small fraction consists of animal studies and clinical trials have been conducted with two compounds (Table 3).

**Table 3:** A summary of selected *in vitro* and animal/clinical studies involving phenylpropanoids

Compound (class)	Mechanism of action	Target pathogen/infection (reference)	Type of study
KIN101 (isoflavone)	Activates interferon regulatory factor IRF-3	Hepatitis C virus, influenza A virus (304)	Cell culture; first small molecule stimulators of the human innate immune system
Baicalein (flavonoid)	Synergy with ciprofloxacin kills ciprofloxacin-resistant bacteria	Ciprofloxacin-resistant methicillin resistant *S. aureus* (305)	*In vitro*; ciprofloxacin = “last resort” antibiotic with increasing resistance
Hymecromone, scoparone (coumarins)	Reactivate latent virus, enable viral clearance by other agents	HIV-1 (306)	Cell culture; HIV-1 reservoir eradication, low cytotoxicity
(-)-Hopeaphenol (tetrameric resveratrol)	Blocks type III secretion systems in Gram-negative bacteria, prevents growth of *Chlamydia trachomatis* (sexually transmitted disease “chlamydia”)	*P. aeruginosa*, *Yersinia pseudotuberculosis* (307)	*In vitro*, cell culture (*Chlamydia*); virulence attenuator
Arctiin (lignin)	Elicits the production of virus-specific antibodies	Influenza A virus (308)	*In vivo*, synergy with approved antiviral (oseltamivir)
23-(*S*)-2-Amino-3-phenylpropanoyl-silybin	Enhanced survival rate of infected mice, 100-fold drop in viral titers in the lungs, modulates inflammatory responses	Influenza A virus (309)	*In vivo*
Silibinin 2',3-di(sodium succinate) or Legalon SIL	Prevents production of hepatitis C virus, elevates anti-inflammatory responses	Hepatitis C virus (310)	*In vivo*, may be sold in Austria, Belgium, Germany, Luxembourg, and Switzerland
Podophyllotoxin (lignan)	Clears anogenital warts with equal efficacy to imiquimod	Human papilloma virus (311)	Randomized clinical trial, wart clearance time about 4 weeks compared with 16 weeks for imiquimod
Bicyclol (lignan)	Inhibition by upregulation of the glycolipid transfer protein leading to arrest of viral replication, reduces liver inflammation by suppression of mitogen-activated protein kinase/nuclear factor κB signaling	Hepatitis B and hepatitis C viruses (312, 313, 314)	Inhibits hepatitis B and hepatitis C viruses without toxic effects, enhances effect of established antivirals; used in China

### Isoflavonoids

Abreu *et al*. explored 22 different isoflavonoids for synergistic effects with approved antibiotics and found that *Cytisus striatus* (Fabaceae) or Portuguese broom contained powerful antibiotic adjuvants, which enabled ciprofloxacin and erythromycin to control MRSA (36). Glabridin acts synergistically with the antifungal fluconazole, to permeabilize cell membranes of *Candida* spp. and *Cryptococcus neoformans* (315). *Dalea formosa* (Fabaceae) produces several isoflavonoids that can synergize with antifungals against *Candida* spp. (316). Efflux pump inhibition by isoflavonoids is considered to underlie both their antibacterial (36) and antifungal (316) effects.

Isoflavones have also been investigated for broad-spectrum activity against RNA viruses (304). In antiviral development, molecules that stimulate host defense are more desirable, since development of resistance is less likely compared with drugs that target specific viral components. Using a high-throughput screening assay, Bedard *et al*. identified a series of isoflavones, the most prominent of which was KIN101, as the first small molecule stimulants of the human innate immune system, which activate the interferon regulatory factor, IRF-3, and enable the elimination of hepatitis C virus (HCV) and IAV infections (304).

### Flavonoids

The Chinese skullcap (*Scutellaria baicalensis*; Lamiaceae) produces the flavonoids baicalein, wogonin, and oroxylin A (317). Baicalein acts synergistically with ciprofloxacin to overcome ciprofloxacin resistance in MRSA strains (305). This is significant considering that ciprofloxacin is a “last resort” antibiotic to which resistance is rising. Baicalein and wogonin are able to kill pathogenic fungi such as *Aspergillus fumigatus, Trichophyton mentagrophytes,* and *T. rubrum* by accelerating ROS production (318).

Chartaceones (dialkylated flavonones) C-F extracted from *Cryptocarya chartacea* (Lauraceae) inhibited the DENV NS5 polymerase, a key enzyme involved in viral replication and showed low micromolar inhibitory concentrations with no toxicity on mammalian cells (319). The flavonoids apigenin, chrysin, and naringenin were shown to suppress CHIKV entry, replication, and virion production (320), whereas baicalein, fisetin, and quercetagetin inhibited CHIKV infection with favorable inhibitory concentrations and low cytotoxicity, with assays suggesting that these compounds affected the expression of viral proteins and viral RNA synthesis (321).

### Coumarins

Coumermycin A1 is a known DNA gyrase B inhibitor that prevents replication of HIV by interfering with the Hsp90 and capsid proteins (322). Unsubstituted coumarin is well regarded as a promising antiviral lead and targets several components including viral polymerases, surface antigens, proteases, and host defense pathways involving mTOR (mammalian target of rapamycin) and NF-κB (323). A major issue in treating viral infections is reservoir eradication; *i.e.*, the elimination of latent viruses in infected cells. The coumarins hymecromone (4-methylumbelliferone) and scoparone reactivate latent reservoirs of HIV-1 with low cytotoxicity, enabling more effective viral clearance by antiretroviral agents (306).

### Chalcones or 1,3-diaryl-2-propen-1-ones

These are biosynthetic intermediates in flavonoid and isoflavonoid metabolism and precursors of aurones formed by polyketide synthase–type enzymes. They are commonly produced in plant families such as the Asteraceae, Leguminosae, and Moraceae (324) and have been used as privileged scaffolds for drug development (325). For example, potent antimalarial activities of conjugates of chalcones with coumarins and chloroquines have also been reported (326, 327). The reaction of their α,β-unsaturated groups with biological nucleophiles, such as the thiol residues of proteins, is considered the underlying mechanism for the seemingly diverse antibacterial, antifungal, antiparasitic, and antiviral activities of chalcones (328).

Chalcones have also been used as probes to identify molecular targets of drug-like molecules *via* activity-based protein profiling (329). 4-Hydroxyderricin extracted from the carrot family plant tomorrow's leaf (*Angelica keiskei*) possesses strong antibacterial activity, including against *S. aureus*. A probe using click chemistry identified seryl-tRNA synthetase as the target of 4-hydroxyderricin (329). Owing to the well-known structures and functions of aminoacyl t-RNA synthetases, they are considered promising targets for antibacterial and antiparasitic drug development (330).

### Aurones

These are 2-benzylidenebenzofuran-3-ones, derived from enzymatic modifications of chalcones and found commonly in flowers of the genera *Cosmos* (family Asteraceae) and *Antirrhinum* (family Plantaginaceae). The aurone scaffold is considered a privileged template for antiparasitic development, and 4,6-dimethoxy substituted aurones show potent antiparasitic activity against the intracellular amastigote form of *L. infantum* with reduced toxicity compared with the reference drug, amphotericin B (331). Synthetic aurone derivatives with basic groups inhibited the chloroquine-resistant W2 strain of *P. falciparum* and accumulated in the digestive vacuole of the parasite (332). HCV inhibitors that allosterically bind the Thumb Pocket I region of the viral RNA-dependent RNA polymerase have been discovered by screening aurones, with candidates showing good selectivity and low toxicity (333). Of interest, replacement of one of the cyclic aurone structures (the B ring) with an indole increased the HCV RNA-dependent RNA polymerase inhibition (334).

### Stilbenes

Resveratrol is a well-known stilbenoid found in many fruits such as grapes (*Vitis vinifera*), raspberry (*Rubus* spp.), and mulberry (*Morus* spp.). It has antiparasitic properties against *Leishmania major* in both the extracellular promastigote and the amastigote form infecting macrophages (335). Resveratrol analogs inhibited several species of *Leishmania*, and their plasma membrane, cell cycle progression, and vacuoles are the suspected targets (336).

Resveratrol units form more complex structures such as dimers or tetramers, which unlike noncovalent protein oligomers are discrete fused and enlarged structures useful for drug discovery. Anigopreissin A is a dimeric resveratrol, which inhibits reverse transcriptase in nevirapine-resistant HIV strains (337). The tetrameric resveratrol (-)-hopeaphenol produced by *Shorea ovalis* (Dipterocarpaceae) is a virulence attenuator that blocks the type III secretion systems of the Gram-negative pathogens *P. aeruginosa* and *Yersinia pseudotuberculosis* and also prevents growth of the intracellular bacterial pathogen *Chlamydia trachomatis* that causes the sexually transmitted disease chlamydia (307). Various resveratrol-derived monomers and dimers are able to kill Gram-positive food spoilage bacteria such as *Listeria monocytogenes*, and mechanistic studies revealed cell membrane damage as the major mode of action (338).

### Anthocyanidins and catechins

The anthocyanidin cyanidin-3-sambubiocide, which is found in black elderberry (*Sambucus* spp.) extract, was shown to inhibit IAVs (339). This compound is a potent inhibitor of sialidase activity, and a combination of experimental and computational studies suggested that it binds the 430-cavity (the region of the active site containing residues 356–364 and 395–432) of the influenza virus neuraminidase (339). This region is a more desirable target than another previously known cavity around residue 150, which is notably absent in some virulent strains such as H1N1 that caused the 2009 “swine flu” pandemic (340). It is promising that cyanidin-3-sambubiocide does not bind near residues Asp151 and Glu119, mutations that cause antiviral resistance (339). In addition, although mutation of the His274 residue to Tyr in the H1N1 neuraminidase confers oseltamivir resistance, a computational analysis found that cyanidin-3-sambubiocide maintains a stronger affinity for either versions and is predicted to be effective against oseltamivir-resistant H1N1 IAV (341).

The anthocyanins delphinidin and epigallocatechin gallate inhibit flaviviruses spread by mosquitoes, such as the WNV, ZIKV, and DENV (342). WNV assembly was inhibited, whereas the infectivity of ZIKV and DENV was suppressed (342). Using molecular docking and mass spectrometry, the anthocyanins cyanidin, delphinidin, and pelargonidin were analyzed for their binding vis-à-vis the 430-cavity (343). These compounds differ only in the number and position of hydroxyl groups; cyanidin and delphinidin, which contain more hydroxyl groups, bind more effectively than pelargonidin with only one hydroxyl group at the 4′ position. The docking results were in agreement with inhibitory effects in the neuraminidase assays (343).

### Phenalenones

These are a family of polyketides that occur mainly in monocots of the Haemodoraceae, Musaceae, Pontederiaceae, and Strelitziaceae families; nearly all genera within Haemodoraceae produce those compounds (344). They are derived from the condensation of malonyl-CoA units with coumaroyl-CoA or cinnamoyl-CoA precursors (345). Phenalenones are considered good sources of antiparasitic compounds and were lead compounds for the synthesis and testing of amino-substituted 1*H*-phenalen-1-ones and analogs containing a tertiary basic nitrogen. These were more active than the standard drug miltefosine against amastigotes of *L. amazonensis* (346). Natural phenalenones were only moderately active against the malarial parasite *P. falciparum*, but synthetic analogs of these PAs were able to kill chloroquine-resistant strains with low micromolar IC_50_ values and negligible cytotoxicity (347).

### Lignans

The greater burdock *Arctium lappa* L. (Asteraceae) contains the glycosylated lignan arctiin and its aglycone precursor arctigenin, which strongly inhibit IAV, and in mouse models, arctiin elicited the production of virus-specific antibodies (308). Arctigenin suppressed replication of IAV, whereas arctiin had synergistic effects with oseltamivir (308). The flavonoid-lignan extract silymarin from the milk thistle (*Silybum marianum*; Asteraceae), contains four derivatives with wide-ranging antiviral activities (348). Silibinin contains a nearly equimolar mixture of two diastereomers, silybin A and silybin B (349). In mice, silibinin 2',3-di (sodium succinate) or Legalon SIL administration prevented production of HCV and elevated anti-inflammatory responses (310). A silybin derivative, 23-(*S*)-2-amino-3-phenylpropanoyl-silybin, enhanced the survival rate of IAV-infected mice and reduced viral titers in the lungs by 100-fold, while modulating a number of inflammatory responses (309).

In a randomized clinical trial, podophyllotoxin, an antiviral lignan from *Podophyllum* species (Berberidaceae), was found to be equally safe and effective compared with the antiviral imiquimod for the treatment of anogenital warts caused by human papilloma virus; however, the clearance time was reduced to 4 weeks compared with 16 weeks for imiquimod (311). In clinical trials, another well-known lignan, bicyclol, effectively inhibited HBV and HCV without adverse effects and enhanced the action of established antivirals (312). Inhibition of HCV was likely through upregulation of the glycolipid transfer protein, leading to arrest of viral replication (313). Furthermore, bicyclol reduced liver inflammation in mice infected by HCV *via* the suppression of MAPK/NF-κB signaling (314).

## Challenges and the way forward

As of 2019, there were 27 antimicrobial peptides derived from various kingdoms of life in clinical and 9 in preclinical development (350). The high proportion of antimicrobial peptides in the *in vitro* testing stages (Table 1) likely reflects the fact that many of them have been recently characterized and have not yet been tested or that formulation and delivery issues were identified. Delivery issues do not preclude their usefulness as structural leads for further optimization. Some plant antimicrobial peptides, however, are also toxic to mammalian cells. Generally, the proteolytic stability of peptides is a major limitation in synthetic therapeutic peptide development, and this necessitates extensive investment in techniques for stabilizing their structures *in vivo* (351). In contrast, many plant defense peptides are inherently stabilized by disulfide bridges and cyclic structures, providing resistance to proteolysis and often membrane-penetrating properties (352). Cyclic antimicrobial peptides lack free amino and carboxyl termini, conferring resistance to exopeptidases, while their increased rigidity, compared with linear peptides, provides resilience against endopeptidases (353). Several antimicrobial peptide classes are tolerant of amino acid substitutions, where their antimicrobial activity is dependent more on their three-dimensional structure than their exact sequences. Given these biochemical advantages, the ease of computational structural modeling, the improvements in peptide synthesis, and the development of heterologous expression tools, plant antimicrobial peptides are now poised for prosperity in antimicrobial drug development.

Alkaloid biosynthesis is common in several plant families (208) and includes many historically marketed compounds, yet their use in recent drug development remains infrequent, limited by their ionization, acidity, and solubility properties (204). Availability of source material is another issue, especially since climate change is accelerating loss of biodiversity (209) and threatening supplies of medicinal plants. Advances in both the chemical synthesis and expansion of biological tools has opened possibilities for the chemical synthesis, semisynthetic modification, and heterologous production of plant compounds. The total synthesis of some terpenoid indole alkaloids has been achieved using catalytic asymmetric reactions and radical cascades (233). However, these are challenging and usually involve multiple procedures, with steadily diminishing yields for each extra step. These disadvantages may be partly overcome, since the heterologous production of certain terpene indole alkaloids, for example, strictosidine, is now possible (60) and hairy root cultures for the production of terpene alkaloids have also been developed (234). Yeast platforms can now heterologously produce tropane alkaloids of medicinal importance (354). In particular, the combination of metabolomics and next-generation sequencing promises to facilitate the elucidation of secondary metabolite pathways in medicinal plants (355). It is reasonable to expect that, with an increased understanding of the biosynthesis of other alkaloids, many more could be mass produced in the near future.

For compounds such as phenylpropanoids (for example, curcuminoids), one of the major challenges is their activity as PAINS (pan-assay interference compounds). PAINS contain promiscuous structural motifs leading to apparent positive readouts in biological assays and cannot be further developed (356). However, closer inspection of predicted PAINS revealed that molecules characterized as PAINS actually contained not only false positives and “false hits,” which react indiscriminately with the target or interfere with the assay procedure and are thus unsuited for further development, but also “true hits,” which can be further optimized using orthogonal assays (356). Some plant secondary metabolites can be ingested in gram quantities and act nonspecifically by altering inflammatory responses *in vivo*, often *via* negative feedback mechanisms (357). In the context of antivirals, molecules that stimulate host defenses in a pathogen-unspecific manner may still be useful, since probable mutations may render highly specific inhibitors ineffective. However, in addition to generic modulation of mammalian antiviral defense mechanisms such as the MAPK pathways (358), some phenylpropanoids also have specific actions against certain viruses. A case in point is the induction of IAV-specific antibodies by arctiin (308).

In fact, in contrast to the conventional drug discovery process that concentrates on a small set of molecules against specific diseases, the adsorption, dispersion, metabolism, and excretion of drugs entail interactions with multiple systems of proteins. Based on the multiple target engagement hypothesis, the Computational Analysis of Novel Drug Opportunities (CANDO) platform creates a molecular interaction signature of drugs and could construct a library of human-ingestible compounds carrying minimal side effects (359). Thus, global proteomics-based approaches may improve the evaluation of compounds showing poly-pharmacology. In addition, unlike the earlier era of “spray and pray” studies, more computational screening efforts involving phytochemicals are coming up, and some of them also include toxicity and adsorption, dispersion, metabolism, and excretion evaluations (360). Efforts are also being directed to redress some of the liabilities of phytochemicals in drug development by applying advances in total synthesis, semisynthesis, and gene cluster manipulation to improve their activity, toxicity, selectivity, or other properties (361).

## Conclusions

Plant defense compounds such as antimicrobial peptides, noncanonical amino acids and their derivatives, alkaloids, sulfur compounds, and phenylalanine/tyrosine-derived compounds possess a wide variety of antibacterial, antifungal, antiparasitic, and antiviral activities. Both isolated compounds and compounds synthesized using their core structures have shown effective and clinically safe biological activity pertaining not only to noncommunicable diseases but also to a variety of infectious diseases. Clinical efficacy has been shown for a number of plant extracts; however, clinical studies of the corresponding isolated active compounds and their molecular mechanisms is underexplored. It should be noted that aspects related to design, evaluation, and assessment of the clinical trials published and cited throughout are outside the scope of this review. Plant natural product chemistry is still an area ripe for discovery of compounds with scientific and medical interest, which can be expected to only increase in the post-genomic era. Plant defense molecules are among the most promising drug candidates, and they provide advantages when compared with large synthetic libraries for facile development of anti-infective drugs that are necessary to combat current and emerging infections and diseases.

## Conflict of interests

The authors declare that they have no conflicts of interest with the contents of this article.
